# Identification of Prognostic Factors and Regulatory Pathways in Porto‐Sinusoidal Vascular Disorder

**DOI:** 10.1111/liv.70898

**Published:** 2026-09-30

**Authors:** Subin Heo, Do Kyung Yoon, Soyeon Shin, Dami Youn, Youngeun Yoo, In Hye Song, Hyo Jeong Kang, Young‐In Yoon, Kyunggon Kim, Seung Soo Lee, Sung Won Chung, Jonggi Choi, Danbi Lee, Ju Hyun Shim, Kang Mo Kim, Young‐Suk Lim, Han Chu Lee, Mark Muthiah, Ho‐Su Lee, Won‐Mook Choi

**Affiliations:** ^1^ Department of Radiology and Research Institute of Radiology, Asan Medical Center University of Ulsan College of Medicine Seoul Korea; ^2^ Department of Biochemistry and Molecular Biology, Brain Korea 21 Project, Asan Medical Center University of Ulsan College of Medicine Seoul Korea; ^3^ Department of Gastroenterology, Liver Center, Asan Medical Center University of Ulsan College of Medicine Seoul Korea; ^4^ Department of Pathology, Asan Medical Center University of Ulsan College of Medicine Seoul Korea; ^5^ Department of Pathology Ewha Womans University College of Medicine Seoul Korea; ^6^ Division of Hepatobiliary Surgery and Liver Transplantation, Department of Surgery, Asan Medical Center University of Ulsan College of Medicine Seoul Korea; ^7^ Department of Digital Medicine, Brain Korea 21 Project, Asan Medical Center University of Ulsan College of Medicine Seoul Korea; ^8^ Department of Medicine, Yong Loo Lin School of Medicine National University of Singapore Singapore Singapore

**Keywords:** IL‐6, liver‐to‐spleen volume ratio, Noncirrhotic portal fibrosis, transcriptomic analysis

## Abstract

**Background and Aims:**

Porto‐sinusoidal vascular disorder (PSVD) is a rare liver condition characterized by specific histological features primarily affecting the hepatic sinusoidal and periportal vasculature, in the absence of cirrhosis. This study aimed to identify prognostic factors and regulatory pathways associated with PSVD by integrating transcriptomic and clinical data.

**Methods:**

A total of 114 PSVD patients were included, with 74 followed longitudinally for liver‐related events. RNA sequencing was performed on 21 liver samples and compared to six histologically normal livers. Associations between clinical parameters, such as liver‐to‐spleen volume ratio (LSVR) and fibrosis stage, with liver‐related events were assessed. Transcriptomic analyses, including co‐expression and cell deconvolution, were conducted based on these parameters.

**Results:**

Among the 114 patients, 32 (28.1%) underwent liver transplantation at diagnosis. LSVR strongly correlated with fibrosis stage, which was significantly associated with liver transplantation (per 1‐stage increase: adjusted OR, 14.88; 95% CI: 3.72–59.58). Over a mean follow‐up of 6.7 years in the longitudinal cohort, 12 patients (16.2%) experienced liver‐related events. LSVR was identified as a significant prognostic marker, with an optimal cut‐off value of 1.33 for predicting liver‐related events. Transcriptomic analysis based on LSVR and fibrosis stage revealed distinct gene expression patterns and cellular changes in PSVD, including shifts in liver sinusoidal endothelial cell (LSEC) distribution, an increased hepatic stellate cell population, and upregulation of IL‐6 signalling, without changes in immune cell composition as the disease progresses.

**Conclusions:**

This study identified LSVR as a novel prognostic marker in PSVD and suggests that IL‐6 trans‐signalling‐induced endotheliopathy in LSECs may play a role in the proinflammatory and fibrotic changes associated with disease progression.

## Key Points

**What is already known on this topic?:**

- Porto‐sinusoidal vascular disorder (PSVD) is a rare liver condition characterized by portal hypertension in the absence of cirrhosis.
- Due to its low incidence, little is known about its prognostic factors and regulatory pathways.

**What this study adds?:**

- In 114 patients with PSVD, the liver‐to‐spleen volume ratio (LSVR), which strongly correlated with the fibrotic burden, was associated with liver‐related events (e.g., liver‐related death, liver transplantation).
- Transcriptomic analysis stratified by LSVR and fibrosis stage suggested that IL‐6 trans‐signalling‐induced endotheliopathy may contribute to the proinflammatory and fibrotic changes that accompany disease progression.

**Abbreviations**
AUROC: area under the receiver operating characteristic curve
CI: confidence interval
CTP: Child‐Turcotte‐Pugh
DEG: differentially expressed genes
FC: fold change
FDR: false discovery rate
FFPE: formalin‐fixed, paraffin‐embedded
GEO: gene expression omnibus
HIV: human immunodeficiency virus
HNL: histologically normal liver
HSC: hepatic stellate cell
IL: interleukin
LIF: leukaemia inhibitory factor
LSEC: liver sinusoidal endothelial cell
LSVR: liver‐to‐spleen volume ratio
LT: liver transplantation
MELD: model for end‐stage liver disease
OR: odds ratio
PSVD: porto‐sinusoidal vascular disorder
ROC: receiver operating characteristic
Selfox: selenium‐enriched diet plus FOLFOX PSVD rat model

## 1 Introduction

Porto‐sinusoidal vascular disorder (PSVD) is a clinicopathological entity characterized by specific histological lesions encompassing the portal venules and sinusoids, in the absence of cirrhosis. While historically known as non‐cirrhotic portal hypertension, the current definition of PSVD encompasses a broader clinical spectrum, including patients who do not yet exhibit signs of portal hypertension [1]. PSVD is associated with diverse underlying conditions, including prothrombotic states, immunological disorders, chronic infections (e.g., human immunodeficiency virus infection), and exposure to specific drugs such as thiopurines or oxaliplatin [2, 3]. While these factors are increasingly recognized, a significant proportion of cases remain idiopathic, with no identifiable systemic trigger. Due to the rarity of the disease and limited awareness, PSVD is often misdiagnosed as cirrhosis, leading to an underestimation of its true prevalence and prognostic impact [1].

The pathophysiology of PSVD remains poorly understood, and current treatments primarily focus on managing complications associated with portal hypertension rather than altering the natural course of the disease. A previous study using biological network analysis of 20 PSVD cases, compared with 21 sex‐ and age‐matched patients with cirrhosis and 13 histologically normal livers (HNLs), reported that PSVD is characterized by dysregulation in pathways related to vascular homeostasis, lipid metabolism, coagulation, and oxidative phosphorylation [4]. However, as this study relied on microarray data for the primary analysis and did not assess transcriptomic changes in relation to disease severity or prognosis, it was limited in providing deeper insights into the dynamic changes in biological pathways as the disease progresses.

Generally, PSVD is associated with a better prognosis than cirrhosis, primarily due to preserved liver function and the slow progression of the disease. However, in some cases, it can lead to decompensation and mortality [5]. The presence of ascites and underlying conditions associated with PSVD such as HIV infection were reported as prognostic factors [6, 7, 8, 9]; however, data on the prognostication of patients with PSVD are very limited. Identifying prognostic factors and regulatory pathways is crucial for discovering new therapeutic targets and improving patient outcomes, particularly in rare diseases like PSVD.

In this longitudinal cohort study of PSVD patients, we integrated transcriptomic and clinical data (i) to identify clinically applicable prognostic factors, including the liver‐to‐spleen volume ratio (LSVR) and fibrotic burden, in a well‐characterized PSVD cohort, and (ii) to delineate the underlying regulatory pathways that may explain disease progression and inform future therapeutic strategies. We identified LSVR as a novel marker for predicting fibrotic burden and liver‐related events, and further found that IL‐6 trans‐signalling‐induced endotheliopathy of periportal liver sinusoidal endothelial cell (LSEC) may serve as a potential driver of inflammation and fibrosis during PSVD progression.

## 2 Methods

### 2.1 Study Population

Patients with a pathological diagnosis of either ‘idiopathic non‐cirrhotic portal hypertension’, ‘hepatoportal sclerosis’, ‘non‐cirrhotic portal fibrosis’, ‘incomplete septal cirrhosis’, or ‘porto‐sinusoidal vascular disease/disorder’ based on pathology reports between April 2005 and August 2023 at Asan Medical Center, and with available liver tissue specimens obtained through either biopsy or liver explant, were evaluated for study eligibility. Two board‐certified liver pathologists (Y.Y., with 8 years of experience and H.J.K., with 18 years of experience) conducted a detailed review of the patients' pathological slides. To ensure diagnostic reliability, biopsy specimens were evaluated for adequacy; only samples with a minimum length of 15 mm and containing at least 10 complete portal tracts were included for histological assessment. The diagnosis of PSVD was confirmed using composite clinical and histological criteria, as outlined in the updated PSVD criteria [10]. Specifically, diagnosis required (1) at least one specific histological feature, (2) at least one specific sign of portal hypertension, or (3) a combination of non‐specific histological changes together with non‐specific signs of portal hypertension (Table S1). Ultimately, 114 patients were included in the study: 82 diagnosed via biopsy and 32 via explant specimens. Among these patients, a longitudinal sub‐cohort of 74 patients was followed to observe liver‐related events, excluding those who had undergone liver transplantation (LT) at baseline (*n* = 32), those for whom the LSVR could not be measured due to prior splenectomy (*n* = 4), and those with a follow‐up period of less than 6 months (*n* = 4). For transcriptomic analysis, 21 PSVD patients from whom sufficient RNA could be extracted from formalin‐fixed, paraffin‐embedded (FFPE) liver tissue were selected and compared with a control group of 6 individuals with HNL who had no history of medication use (e.g., oxaliplatin, azathioprine) or conditions that could cause structural changes in the liver.

This study was approved by the Institutional Review Board of Asan Medical Center (IRB number: 2021‐0653) and was conducted in accordance with both the Declaration of Helsinki and the Istanbul Protocol. Due to the retrospective nature of the study, the requirement for informed consent was waived.

### 2.2 Covariates and Outcomes

Baseline characteristics, including age, sex, underlying diseases, and the history of any events related to portal hypertension (e.g., variceal bleeding, ascites, or encephalopathy), were recorded. Laboratory values, including aspartate transaminase, alanine transaminase, albumin, total bilirubin, platelet counts, creatinine, white blood cell count, haemoglobin, and international normalized ratio, were collected. Child‐Turcotte‐Pugh (CTP) scores and Model for End‐Stage Liver Disease (MELD) scores were calculated using clinical data. Liver stiffness values from transient elastography were obtained for patients who had results available within 3 months prior to diagnosis. The METAVIR scoring system was utilized to assess the severity of liver fibrosis.

For the volumetric evaluation of the liver and spleen, abdominal CT scans obtained during the portal venous phase within 3 months prior to liver biopsy or explantation were utilized. A deep learning algorithm for automated liver and spleen segmentation, implemented in a web‐based DICOM viewer software (GoDCSS; SmartCareworks Inc.), was used to process the CT images. This algorithm automatically segmented the liver and spleen and calculated their volumes (cm^3^) by summing the consecutive areas of the organs in each slice and multiplying them by the slice thickness (Figure S1). A board‐certified radiologist (S.H., with 8 years of experience), who was blinded to the clinicopathologic data and patient outcomes, reviewed the deep learning‐generated segmentation results and corrected any segmentation errors. The LSVR was calculated by dividing the liver volume by the spleen volume.

For the longitudinal follow‐up of clinical outcomes, patients were monitored from the index date, defined as the date of PSVD diagnosis, until the occurrence of liver‐related events (i.e., liver‐related death or LT) or until the last follow‐up date (April 10, 2026).

### 2.3 RNA Sequencing Analysis

RNAs were extracted from FFPE tissues of patients with PSVD and HNL using the RNeasy FFPE Kit (QIAGEN GmbH, Hilden, Germany) according to the manufacturer's instructions. RNA sequencing data were processed using RNA‐SeQC for gene expression quantification, with quality control conducted using FastQC and MultiQC. Reads were trimmed with Cutadapt and subsequently mapped to the GRCh38 reference genome using STAR. Uniquely mapped reads were selected, and gene expression was quantified in transcripts per million (TPM). Differentially expressed genes (DEGs) were identified using DESeq2 after filtering out low‐expression genes, with cutoff criteria set at|log_2_ Fold Change (log_2_FC)| > 2.0 and a false discovery rate (FDR) < 0.05 [11]. DEGs were visualized through volcano plots and heatmaps, and gene set enrichment analysis was performed with clusterProfiler using MSigDB Hallmark gene sets [12]. Cell subset proportions were estimated using CIBERSORTx [13], with single‐cell RNA‐seq data from the Gene Expression Omnibus (GEO; GSE115469) serving as the reference [14]. Co‐expression network analysis was performed using weighted gene co‐expression network analysis [15], with module identification based on topological overlap. PSVD‐related modules were correlated with clinical data, and key pathways were identified through Ingenuity Pathway Analysis. Detailed information on the experimental methods is provided in Supporting Information.

The raw data from the RNA sequencing conducted in this study have been submitted to the NCBI GEO under the accession number GSE279028. This study used previously published gene expression datasets (GSE77627: microarray [4]; GSE229380: RNA‐seq [16]) for validation and comparison.

### 2.4 Immunofluorescence Staining

For immunofluorescence, FFPE tissue sections were used. Tissue sections were deparaffinized in xylene and rehydrated in water. Antigen retrieval was performed using proteinase K (P2308; Sigma‐Aldrich, USA). The primary antibodies used for immunofluorescence staining were mouse anti‐IL‐6 (MA‐45069; Invitrogen, USA), mouse anti‐STAT3 (9139s, Cell Signalling, USA), and rabbit anti‐CD36 (14347s; Cell Signalling, USA). Tissues were incubated with the primary antibodies for 16 h at 4°C. Secondary antibodies were used at a 1:200 dilution: anti‐mouse Alexa Fluor 488 (ab150105; Abcam, UK) and anti‐rabbit Alexa Fluor 594 (ab150080, Abcam, UK). Nuclei were counterstained and mounted using Fluoroshield with DAPI (F6057, Sigma‐Aldrich, USA). Fluorescent confocal images of human liver tissues double‐ or triple‐stained for IL‐6, STAT3, CD36, and DAPI were acquired using a ZEISS LSM 880 confocal microscope and ZEN software. Immunofluorescence was performed on 10 liver samples (3 HNLs, 3 early PSVD, and 4 cirrhosis), with five non‐overlapping fields analysed per sample.

### 2.5 Statistical Analysis

Continuous variables were compared using either the Student's *t*‐test or the Mann–Whitney *U* test, depending on the distribution of the data. Categorical variables were compared using the chi‐square test or Fisher's exact test. The Kruskal–Wallis test was applied for comparisons involving three groups. The association between LT and various prognostic parameters (LSVR, fibrosis stage, MELD score, and CTP score) was evaluated through univariate and multivariable logistic regression analyses. Univariate Cox regression analysis was conducted to identify risk factors associated with liver‐related events in the longitudinal cohort. The cut‐off value for LSVR was determined using receiver operating characteristic (ROC) analysis and the Youden index. An LSVR cut‐off of 1.33 was applied to dichotomize patients into two groups, reflecting both clinical interpretability and ROC‐based performance. The 95% confidence interval for the area under the receiver operating characteristic curve (AUROC) was estimated using the DeLong method. Internal validation of the model discrimination was performed by bootstrap resampling (2000 resamples) with optimism correction after Harrell, yielding an optimism‐corrected AUROC; the same bootstrap procedure was applied to the Youden‐derived cut‐off to obtain optimism‐corrected estimates of its sensitivity and specificity. Liver‐related event‐free survival was evaluated using the Kaplan–Meier method and compared with the log‐rank test based on LSVR and fibrosis stage. Pearson's correlation coefficient was used to examine relationships between module expression, cell type proportions, and prognostic parameters. All statistical analyses were conducted using R version 4.1.2 (R Foundation for Statistical Computing, Vienna, Austria; http://cran.r‐project.org/). All tests were two‐sided and *p* < 0.05 was considered to denote statistical significance.

## 3 Results

### 3.1 Patient Characteristics

The baseline characteristics of the study patients are summarized in Table 1. A total of 114 patients were included, with a mean age of 46.7 ± 19.2 years. Among them, 32 patients (28.1%) underwent LT at the time of diagnosis. Of all patients, 63 (55.3%) were male, and 46 (40.7%) had portal vein thrombosis. Prothrombotic disorders (21.9%) and haematological disorders (5.3%) were the most common associated conditions. Exposure to potentially hepatotoxic drugs, such as thiopurines or oxaliplatin, was identified in 3.5% of patients. The median MELD score was 9 (interquartile range [IQR], 7–12), and the median LSVR was 1.67 (IQR, 0.88–3.19). Of the 114 patients, 65 (57.0%) presented with specific histological signs (58 with obliterative portal venopathy, 38 with incomplete septal fibrosis, and 21 with nodular regenerative hyperplasia; Table S1), and 93 (81.6%) presented with specific signs of portal hypertension (81 with oesophageal varices, 36 with variceal bleeding, and 62 with porto‐systemic collaterals at imaging). Ten patients (8.8%) without either a specific histological sign or a specific sign of portal hypertension were diagnosed with PSVD based on the presence of both the non‐specific histological criteria and the non‐specific sign of portal hypertension.

**TABLE 1:** Baseline characteristics of the study patients.

Characteristics	Entire cohort (*n* = 114)	No transplantation (*n* = 82)	Transplantation (*n* = 32)	*p*
Age, mean ± SD, year	46.7 ± 19.2	45.8 ± 21.2	49.0 ± 12.6	0.43
Male sex, *n* (%)	63 (55.3)	47 (57.3)	16 (50.0)	0.62
Specific signs of portal hypertension				
Varix, *n* (%)	81 (71.1)	53 (64.6)	28 (87.5)	0.03
Variceal bleeding, *n* (%)	36 (31.6)	25 (30.5)	11 (34.4)	0.86
Porto‐systemic collaterals at imaging	63 (55.3)	40 (48.8)	23 (71.9)	0.03
Non‐specific signs of portal hypertension				
Platelets < 150 × 10^3^/μL	81 (71.1)	54 (65.9)	27 (84.4)	0.05
Ascites, *n* (%)	39 (34.2)	18 (22.0)	21 (65.6)	< 0.001
Spleen size ≥ 13 cm in largest axis	64 (56.1)	38 (46.3)	26 (81.3)	< 0.001
Encephalopathy, *n* (%)	7 (6.1)	4 (4.9)	3 (9.4)	0.64
Portal vein thrombosis, *n* (%)	46 (40.7)	23 (28.4)	23 (71.9)	< 0.001
Chronic HBV infection, *n* (%)	3 (2.6)	1 (1.2)	2 (6.2)	0.39
Associated conditions/drugs				
Immunological disorder, *n* (%)	4 (3.5)	4 (4.9)	0 (0.0)	0.48
Haematological disorder, *n* (%)	6 (5.3)	4 (4.9)	2 (6.2)	> 0.99
Prothrombotic disorder, *n* (%)	25 (21.9)	19 (23.2)	6 (18.8)	0.79
Drugs, *n* (%)	4 (3.5)	4 (3.5)	4 (3.5)	0.48
Genetic disorder, *n* (%)	1 (0.9)	1 (1.2)	0 (0.0)	> 0.99
HIV infection, *n* (%)	1 (0.9)	1 (1.2)	0 (0.0)	> 0.99
Laboratory values				
AST, median (IQR), IU/L	26 (21, 35)	26 (21, 36)	24 (20, 34)	0.32
ALT, median (IQR), IU/L	19 (13, 26)	21 (14, 34)	16 (12, 21)	0.03
Albumin, median (IQR), g/dL	3.5 (3.0, 3.9)	3.6 (3.1, 3.9)	3.3 (2.9, 3.6)	0.05
Total bilirubin, median (IQR), mg/dL	0.8 (0.6, 1.3)	0.8 (0.5, 1.1)	1.3 (0.8, 2.4)	0.001
Platelets, median (IQR), ×10^3^/μL	100 (54, 169)	107 (70, 196)	50 (41, 88)	0.006
INR, median (IQR)	1.16 (1.05, 1.31)	1.12 (1.03, 1.20)	1.33 (1.18, 1.52)	< 0.001
Creatinine, median (IQR), mg/dL	0.7 (0.6, 0.9)	0.7 (0.6, 0.9)	0.7 (0.5, 0.9)	0.52
White blood cells, median (IQR), ×10^3^/μL	4.0 (3.0, 5.0)	4.2 (3.3, 5.8)	3.4 (2.6, 4.1)	0.15
Haemoglobin, median (IQR), g/dL	10.9 (9.1, 13.1)	11.9 (9.5, 13.2)	9.6 (8.3, 11.0)	0.001
CTP class, *n* (%)				
A	70 (61.4)	61 (74.4)	9 (28.1)	< 0.001
B	38 (33.3)	19 (23.2)	19 (59.4)	
C	6 (5.3)	2 (2.4)	4 (12.5)	
MELD score, mean ± SD	9 (7, 12)	8 (7, 9)	12 (9, 14)	< 0.001
MELD‐Na score, mean ± SD	10 (8, 13)	9 (8, 11)	15 (11, 16)	< 0.001
Liver stiffness, median (IQR), kPa^a^	7.6 (4.9, 11.6)	7.7 (5.0, 11.7)	6.4 (4.9, 10.3)	0.48
Liver volume, median (IQR), cm^3^ ^a^	1054 (796, 1275)	1086 (869, 1284)	886 (679, 1130)	0.06
Spleen volume, median (IQR), cm^3^ ^a^	604 (316, 1049)	450 (278, 728)	1089 (684, 1495)	< 0.001
Liver‐to‐spleen volume ratio, median (IQR)^a^	1.67 (0.88, 3.19)	2.05 (1.31, 3.80)	0.83 (0.52, 1.10)	< 0.001
METAVIR fibrosis score, *n* (%)				
F0	41 (36.0)	40 (48.8)	1 (3.1)	< 0.001
F1	40 (35.1)	34 (41.5)	6 (18.8)	
F2	23 (20.2)	7 (8.5)	16 (50.0)	
F3	10 (8.8)	1 (1.2)	9 (28.1)	

Abbreviations: ALT, alanine aminotransferase; AST, aspartate aminotransferase; CTP, Child‐Turcotte‐Pugh; HBV, hepatitis B virus; INR, international normalized ratio; IQR, interquartile range; MELD, model for end‐stage liver disease; SD, standard deviation.

^a^ Missing data on liver stiffness for 61 patients, and on liver, spleen volume, and liver‐to‐spleen volume ratio for 4 patients.

There were significant differences in baseline characteristics between the LT and non‐LT groups. The LT group had a significantly higher proportion of patients with significant fibrosis (F2–3) (78.1%) compared to the non‐LT group (9.7%). The LT group also had a significantly lower LSVR (0.83 vs. 2.05, *p* < 0.001) and a significantly higher MELD score (12 vs. 8, *p* < 0.001). In the entire cohort, LSVR showed a strong negative correlation with both fibrosis stage (*r* = −0.32) and MELD score (*r* = −0.46) as well as a strong positive correlation with platelet count (*r* = 0.79) (Figure 1).

**FIGURE 1:**
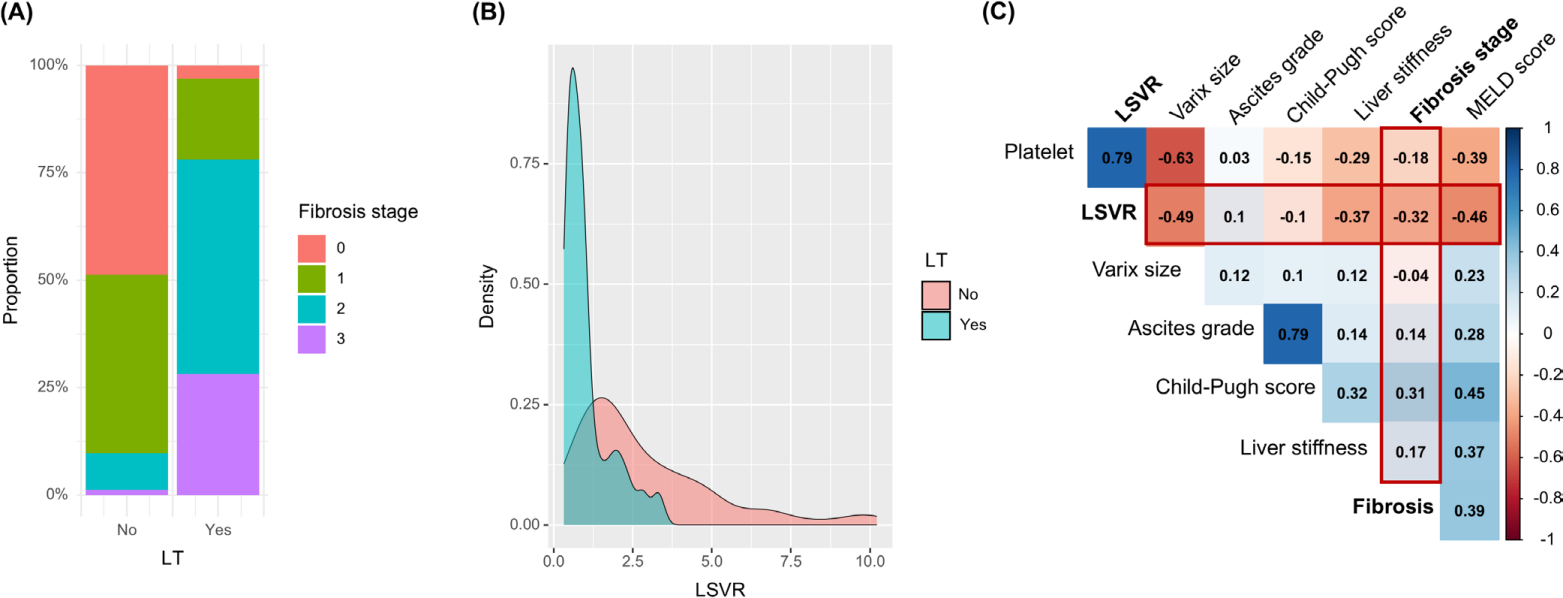
Differences in clinical indicators based on LT status and their correlations. (A) Distribution of METAVIR fibrosis stages by LT status, (B) density plot of LSVR based on LT status, and (C) correlation matrix of clinical indicators representative of portal pressure or liver function, showing Pearson correlation coefficients (*r*). LSVR, liver‐to‐spleen volume ratio; LT, liver transplantation; MELD, model for end‐stage liver disease.

### 3.2 Clinical Parameters Associated With Liver Transplantation

Univariate logistic regression analysis revealed that LSVR (odds ratio [OR], 0.28; 95% confidence interval [CI], 0.14–0.54; *p* < 0.001) and fibrosis stage (per 1‐stage increase: OR, 9.25; 95% CI: 4.09–20.95; *p* < 0.001) were significantly associated with LT. Additionally, the MELD score (OR, 1.26; 95% CI: 1.11–1.43; *p* < 0.001) and the CTP score (per 1‐point increase: OR, 1.86; 95% CI: 1.37–2.52; *p* < 0.001) were also significantly associated with LT (Table 2).

**TABLE 2:** Association between various parameters and the risk of liver transplantation.

Variables	Liver‐to‐spleen volume ratio		Fibrosis stage		MELD score		CTP score	
	OR (95% CI)	*p*	OR (95% CI)	*p*	OR (95% CI)	*p*	OR (95% CI)	*p*
Model 1^a^	0.28 (0.14–0.54)	< 0.001	9.25 (4.09–20.95)	< 0.001	1.26 (1.11–1.43)	< 0.001	1.86 (1.37–2.52)	< 0.001
Model 2^b^	0.17 (0.07–0.39)	< 0.001	10.07 (4.34–23.35)	< 0.001	1.27 (1.12–1.45)	< 0.001	1.89 (1.37–2.60)	< 0.001
Model 3^c^	0.25 (0.11–0.56)	< 0.001	11.20 (4.03–31.13)	< 0.001	1.25 (1.08–1.45)	0.003	1.55 (1.06–2.26)	0.02
Model 4^d^	—	—	14.88 (3.72–59.58)	0.005	1.14 (0.98–1.34)	0.10	1.56 (0.93–2.59)	0.09

Abbreviations: CI, confidence interval; CTP, Child‐Turcotte‐Pugh; MELD, model for end‐stage liver disease; OR, odds ratio.

^a^ Model 1: Unadjusted.

^b^ Model 2: Age and sex‐adjusted.

^c^ Model 3: Ascites and portal vein thrombosis‐adjusted.

^d^ Model 4: Liver‐to‐spleen volume ratio, ascites, and portal vein thrombosis‐adjusted.

The association of four clinical parameters (LSVR, fibrosis stage, MELD score, and CTP score) with the risk of LT was analysed using various combinations of relevant variables based on the results of univariate analysis (Table S2). All four factors remained significantly associated with LT when adjusted for age and sex (*p* < 0.001 for all) and when adjusted for ascites and portal vein thrombosis (*p* < 0.05 for all). However, when adjusted for LSVR, ascites, and portal vein thrombosis, only fibrosis stage (per 1‐stage increase: adjusted OR, 14.88; 95% CI: 3.72–59.58; *p* = 0.005) remained significantly associated with LT (Table 2).

### 3.3 Risk Factors for Liver‐Related Events in the Longitudinal Cohort

The baseline characteristics of the 74 patients in the longitudinal cohort are presented in Table S3. During a mean follow‐up of 6.7 years, 12 patients (16.2%) experienced liver‐related death or underwent LT. In the univariate analysis of risk factors for liver‐related death or LT, LSVR had a hazard ratio of 0.37 (95% CI, 0.15–0.90), with statistical significance (*p* = 0.03) (Table S4). Other clinical parameters, including CTP class, MELD score, and fibrosis stage, were not significantly associated with liver‐related death or LT.

The area under the ROC curve (AUROC) analysis for LSVR in predicting liver‐related death or LT yielded an AUROC of 0.818 (95% CI: 0.687–0.902). In bootstrap internal validation (2000 resamples), the optimism‐corrected AUROC was 0.818, indicating stable discrimination despite the limited number of events. At an LSVR cut‐off of 1.33, the sensitivity was 66.7% and the specificity was 80.6%. Patients with an LSVR > 1.33 demonstrated a significantly better event‐free survival compared to those with an LSVR ≤ 1.33 (*p* = 0.004). When stratified by fibrosis stage, the difference in event‐free survival between the stages was not significant (*p* = 0.59; Figure 2).

**FIGURE 2:**
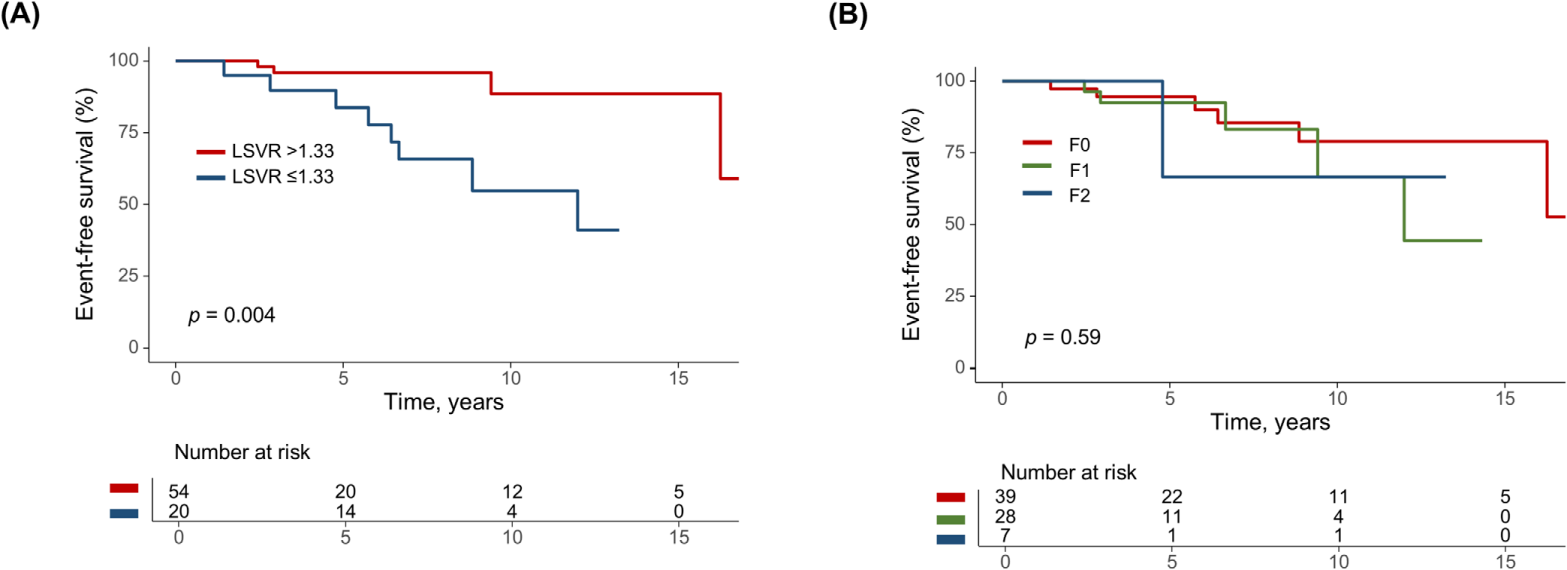
Event‐free survival of patients based on baseline LSVR and fibrosis stage in the longitudinal cohort. (A) event‐free survival based on baseline LSVR and (B) event‐free survival based on baseline fibrosis stage. LSVR, liver‐to‐spleen volume ratio.

### 3.4 Transcriptomic and Cell Composition Changes of PSVD

To investigate the regulatory pathways in PSVD throughout disease progression, we conducted RNA sequencing on 21 PSVD patients (compared with the 93 patients not selected for sequencing, the 21 sequenced patients had more advanced disease, including a higher fibrosis stage, lower LSVR, lower platelet count, and higher MELD score; Table S5) and six HNL controls (Table S6), utilizing prognostic parameters from a cohort study. Principal component and heatmap analyses revealed distinct patterns of DEGs, identifying a total of 1229 DEGs between the two groups (Figure 3A, Table S7, and Figure S2). The most significantly differentially expressed genes were *CIDEC* (log_2_FC = 5.81, FDR = 3.47 × 10^−8^) and *GFI1B* (log_2_FC = 7.18, FDR = 1.14 × 10^−7^). Pathway analysis highlighted seven enriched gene sets in PSVD, including angiogenesis, tumour necrosis factor‐α signalling, inflammatory response, epithelial‐mesenchymal transition, and myogenesis, alongside downregulated metabolic pathways compared to HNL (Figure 3B and Table S8), similar to those identified from the previous datasets [4, 16]. Cell deconvolution analysis revealed increased periportal LSECs and hepatic stellate cells (HSCs), and decreased central venous LSECs in PSVD (*p* < 0.05 for all; Figure 3C,D). In the context of disease progression, periportal LSECs increased and central venous LSECs decreased as LSVR declined, indicating LSEC redistribution as disease advances. While both HSCs and periportal LSECs increased with advancing fibrosis, the patterns across fibrosis stages differed: LSEC changes were evident across disease stages, whereas HSC expansion was most prominent in advanced fibrosis (Figure 3E,F). Ficolin 2 (*FCN2*), a marker gene for central venous LSECs [14], emerged as a differentially expressed gene that decreased with disease progression (Figure S3), suggesting its potential as a biomarker for PSVD progression. Other cell types, including immune cells, showed no significant changes (Figure 3C and Figure S4).

**FIGURE 3:**
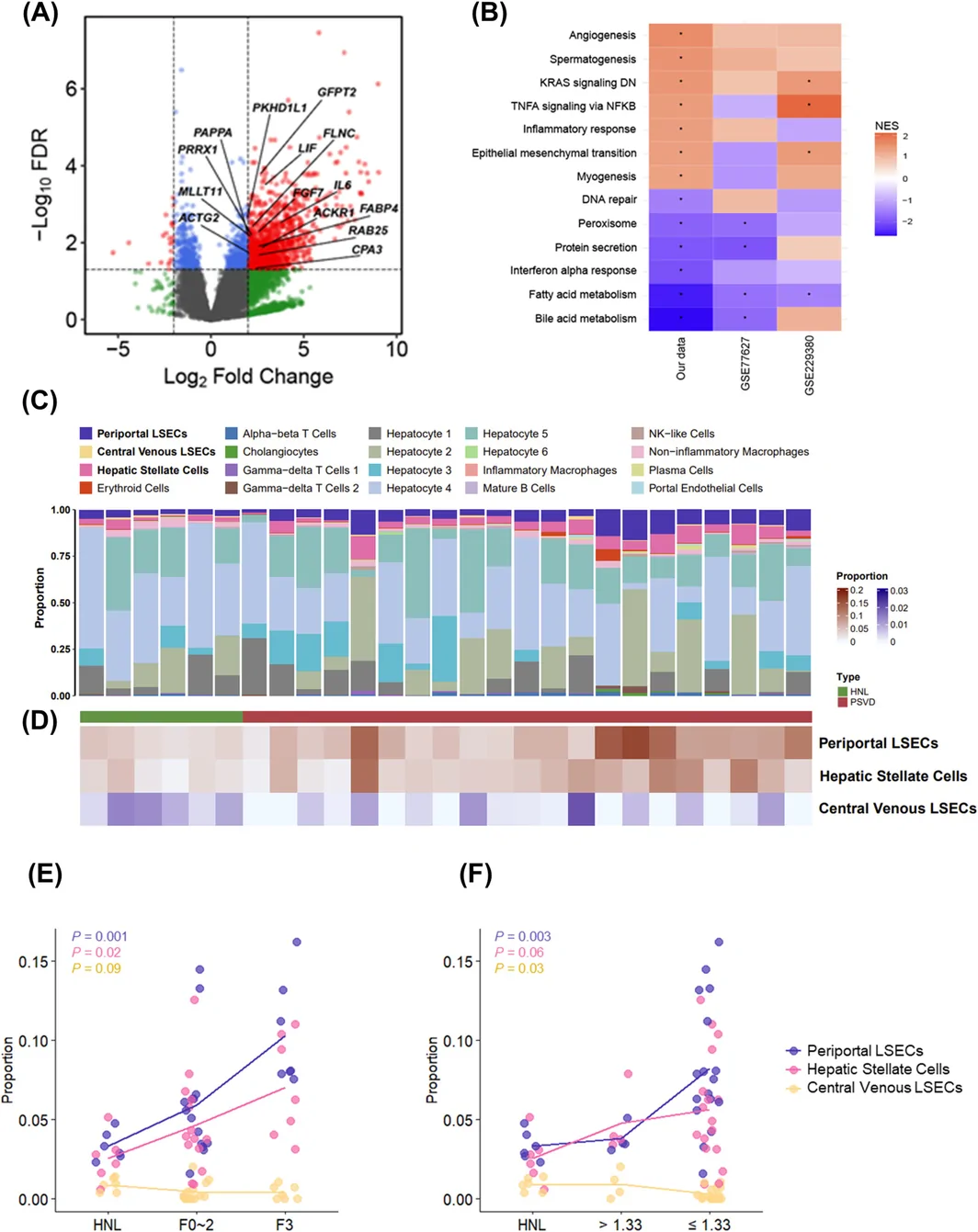
Transcriptomic and cellular landscape of PSVD. (A) Differential gene expression analysis between PSVD patients and HNL subjects. Red dots on the volcano plot highlight significantly up‐ and down‐regulated genes (log_2_FC > 2, FDR < 0.05), with labelled genes overlapping between the PSVD vs. HNL DEGs and the co‐upregulated darkturquoise module. (B) GSEA results in comparison with two public datasets: GSE77627 (PSVD vs. HNL) and GSE229380 (Selfox vs. Control). The heatmap displays NES across all three datasets, with pathways showing FDR < 0.05 marked by an asterisk (*). (C) Relative proportions of liver cell types based on CIBERSORTx deconvolution. (D) Variations in the proportions of periportal LSECs, central venous LSECs, and HSCs between PSVD and HNL. (E) Dot plots showing changes in the proportions of periportal LSECs, central venous LSECs, and HSCs across fibrosis stages (HNL, *n* = 6; F0–F2, *n* = 14; F3, *n* = 7). Each dot represents an individual sample, and lines connect the group‐wise mean values. Statistical significance was assessed using the Kruskal–Wallis test. (F) Dot plots showing changes in the proportions of periportal LSECs, central venous LSECs, and HSCs across LSVR subgroups (HNL, *n* = 6; LSVR > 1.33, *n* = 4; ≤ 1.33, *n* = 17). Each dot represents an individual sample, and lines connect the group‐wise mean values. Statistical significance was assessed using the Kruskal–Wallis test. DEG, differentially expressed genes; FC, fold change; FDR, false discovery rate; GSEA, Gene Set Enrichment Analysis; HNL, histologically normal liver; HSCs, hepatic stellate cells; LSECs, liver sinusoidal endothelial cells; LSVR, liver‐to‐spleen volume ratio; NES, normalized enrichment score; PSVD, porto‐sinusoidal vascular disorder; Selfox, selenium‐enriched diet plus FOLFOX PSVD rat model.

### 3.5 IL‐6 Signalling as a Potential Key Regulatory Pathway in PSVD

Co‐expression analysis identified a significant gene module, termed the darkturquoise module (*r* = 0.52), which correlated strongly with both fibrosis stage and LSVR (Figure 4A,B, Table S9, and Figure S5). Pathway analysis of this module revealed enrichment in processes related to smooth muscle contraction, cardiac hypertrophy, interleukin (IL)‐6, IL‐4, IL‐13 signalling, and wound healing, indicating roles in vascular remodelling and inflammation (Table S10). Fourteen genes overlapped between the DEGs in PSVD vs. HNL and this module. The expression trends of these genes showed distinct patterns across LSVR and fibrosis (Figure 4C). Subgroup analysis based on LSVR and fibrosis stage identified seven genes with significant differential expression, including IL6, between F0–F2 vs. F3 or LSVR ≤ 1.33 vs. > 1.33 groups (Figure 4D, Table S7, and Figure S6). The inclusion of *IL6* suggests the IL‐6 signalling pathway's involvement in disease progression. In addition, Leukaemia Inhibitory Factor (*LIF*) and Cardiotrophin‐Like Cytokine Factor 1 (*CLCF1*), among 24 IL‐6 pathway‐related genes, were differentially expressed between PSVD and HNL samples (Figure 4D), further linking these pathways to disease progression. Gene set enrichment and single‐sample pathway analyses further supported that the PSVD‐associated transcriptomic signature (vascular/inflammatory activation coupled with metabolic/immune suppression) is consistent across fibrosis stages and LSVR categories. The stratified subgroups were, however, small (F0–F2, *n* = 14; F3, *n* = 7; LSVR > 1.33, *n* = 4; ≤G 1.33, *n* = 17) (Figures S7 and S8). When compared with datasets from Hernández‐Gea et al. [4, 16] and the selenium‐enriched diet plus FOLFOX PSVD rat model (Selfox) that mimics key features of early‐stage PSVD, only two genes, *IL6* and *ANKRD1,* were identified as significant DEGs across all three datasets (Figure 4E, and Tables S7, S11, and S12). This was accompanied by consistent upregulation of *IL6ST* (gp130)‐dependent cytokines, including *LIF* and *OSM*, and concurrent downregulation of IL‐6 receptors, suggesting activation of IL‐6 signalling and a potential negative feedback loop via receptor suppression (Figure S9). This finding was further validated by immunofluorescence staining of human liver tissues. While IL‐6 expression progressively increased from HNL to early PSVD and cirrhosis, STAT3 co‐localized most prominently with CD36, a marker of periportal LSECs in zone 1, [17] in early PSVD (Figure 4F), suggesting that IL‐6 signalling in periportal LSECs may act as a distinct early driver of PSVD progression.

**FIGURE 4:**
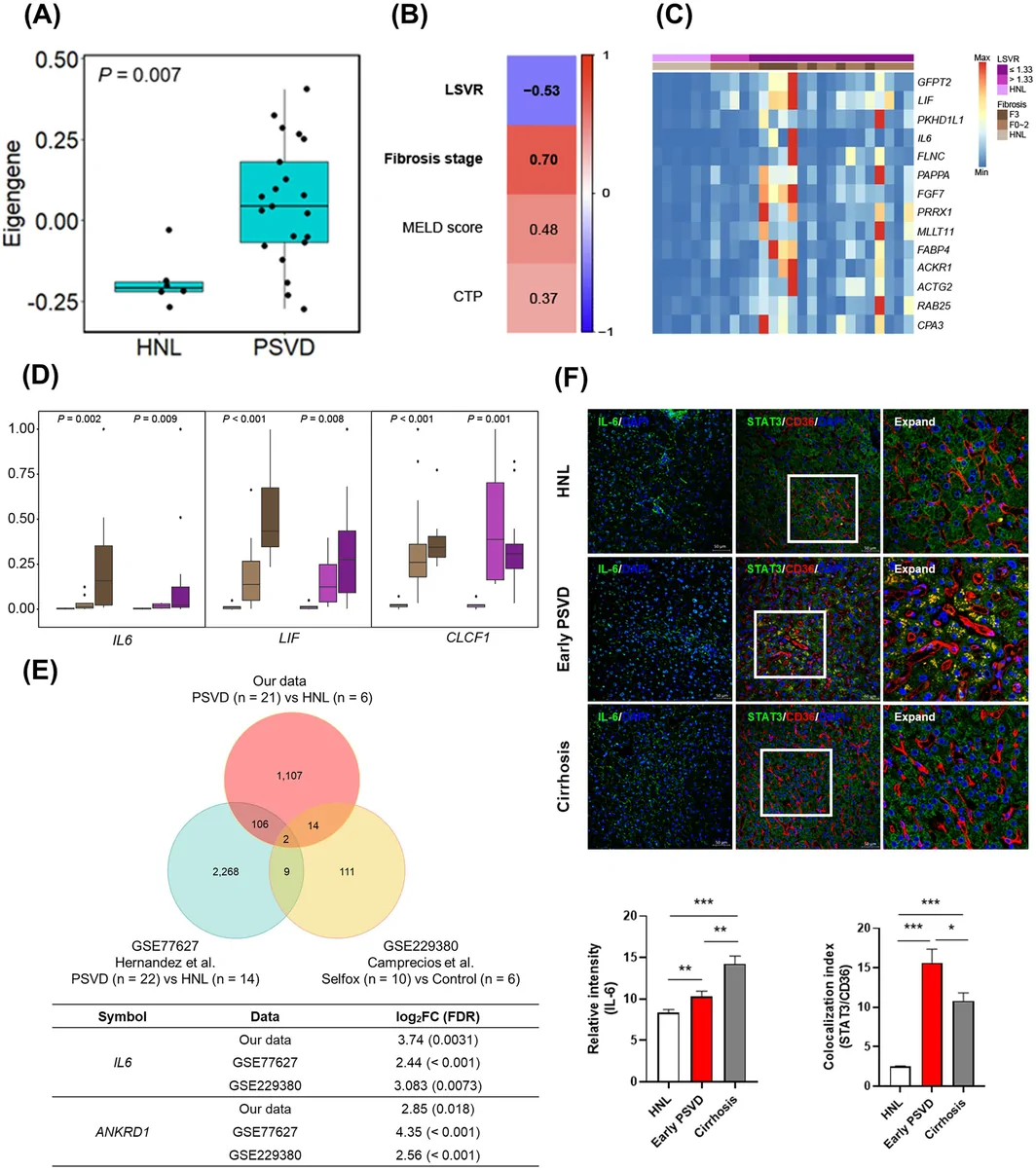
IL‐6 trans‐signalling‐induced endotheliopathy in LSECs as a potential key regulatory pathway in PSVD. (A) Identification of the PSVD‐associated darkturquoise gene module via co‐expression network analysis. (B) Correlations of this module with LSVR, fibrosis stage, MELD score, and CTP score, showing Pearson correlation coefficients (*r*). (C) Heatmap showing expression patterns of 14 overlapping genes in the darkturquoise module between PSVD and HNL. (D) Differential expression of *IL6* and *LIF* between F0–F2 (*n* = 14) and F3 stages (*n* = 7), or between LSVR > 1.33 (*n* = 4) and LSVR ≤ 1.33 (*n* = 17). (E) Comparison of DEGs across three independent transcriptomic datasets. (F) Representative immunostainings of IL‐6, STAT3, and CD36 in liver tissue sections of HNL (*n* = 3), early PSVD (*n* = 3), and cirrhosis patients (*n* = 4). Data are presented as mean ± SEM. *p < 0.05, **p < 0.01, ***p < 0.001. Scale bars, 50 μm. CTP, Child‐Turcotte‐Pugh; DEG, differentially expressed genes; FDR, false discovery rate; HNL, histologically normal liver; log_2_FC, log_2_ fold change; LSECs, liver sinusoidal endothelial cells; LSVR, liver‐to‐spleen volume ratio; PSVD, porto‐sinusoidal vascular disorder; Selfox, selenium‐enriched diet plus FOLFOX PSVD rat model; SEM, standard error of the mean.

## 4 Discussion

Due to the rarity of the disease and the lack of awareness even among experts, our understanding of the pathogenesis, underlying regulatory pathways, and prognostic factors of PSVD has remained limited. In this large cohort of PSVD patients, our comprehensive analysis of transcriptomic and clinical data, ranging from cross‐sectional to longitudinal studies, provides novel insights into the pathogenesis of PSVD. We identified potential biological processes associated with disease progression and their implications for prognosis and therapeutic intervention.

One of the key findings of this study is the role of the LSVR as a prognostic marker for liver‐related events, including LT and liver‐related death. LSVR is a new imaging‐based biomarker for diffuse liver diseases. Its earlier application was constrained by the labor‐intensive process of manual organ segmentation in cross‐sectional imaging. However, recent advancements in deep learning algorithms [18, 19] have made the assessment of LSVR significantly more feasible. LSVR has been studied extensively in the context of cirrhosis, where disease progression leads to liver atrophy and increased spleen volume, resulting in a negative correlation between LSVR and disease severity [20, 21, 22]. Studies have shown that LSVR is a strong predictor of decompensation, liver‐related death, and the need for LT in patients with cirrhosis [23, 24]. We hypothesized that a similar association might exist in PSVD; however, given the unique volumetric changes in PSVD, where liver volume changes are more subtle due to a lower fibrotic burden, while spleen enlargement is much more pronounced due to elevated portal pressure, [25] the prognostic role of LSVR in PSVD requires separate validation.

Our study is the first to quantitatively assess liver and spleen volumes in PSVD, revealing that patients who required LT had significantly lower baseline LSVR. Although the 1.33 cut‐off was derived using the Youden index and evaluated in the same cohort, and only 12 liver‐related events occurred, meaning that the reported sensitivity and specificity should be considered preliminary pending external validation, LSVR nevertheless demonstrated favourable discrimination for liver‐related events in the ROC analysis, supporting its potential as a prognostic marker in PSVD. Indeed, in the longitudinal analysis, prognosis was well‐differentiated when grouped based on LSVR, with a significantly higher risk of liver‐related events in those with an LSVR ≤ 1.33. In contrast, prognostication based on the fibrosis stage was ineffective, likely due to its subjectivity and inability to represent the entire liver (i.e., sampling error). Additionally, while the risk of liver‐related events is continuous, the fibrosis stage consists of distinct categories, rendering it less effective than LSVR in capturing prognosis. Given its non‐invasive nature and potential for tracking disease progression over time, particularly in comparison to liver biopsy, [23] LSVR shows considerable promise as an imaging‐based biomarker in PSVD, with significant clinical implications.

Our study offers important insights into PSVD pathogenesis by integrating clinical prognostic markers (LSVR and fibrotic burden) with stratified transcriptomic analyses—an approach not previously attempted in PSVD—identifying IL‐6 signalling as central to disease progression. Cell deconvolution revealed an expansion of periportal LSECs and HSCs alongside a reduction in central venous LSECs, while immune cell composition remained largely preserved. Although only two genes (*IL6* and *ANKRD1*) overlapped across the three technically heterogeneous transcriptomic datasets (microarray on fresh tissue, RNA‐seq on human FFPE, and RNA‐seq on rat tissue), meaning that the IL‐6 finding should be interpreted as consistent across independent datasets rather than as formal cross‐validation, co‐expression analysis nevertheless highlighted IL‐6 signalling as a key inflammatory pathway, a finding also observed in datasets from Hernández‐Gea et al. [4] and the Selfox rat model recapitulating early‐stage PSVD [16]. Notably, despite an unchanged immune cell composition, IL‐6 upregulation was detectable even in fibrosis‐free datasets, suggesting that chronic IL‐6 exposure is associated with both LSEC capillarization and HSC activation. The shift toward periportal LSECs, together with co‐localization of phosphorylated STAT3 with CD36^+^ periportal LSECs (zone 1) in early PSVD but not in controls, points to IL‐6 trans‐signalling–induced endotheliopathy as an early pathogenic mechanism and identifies periportal LSECs as a likely site of IL‐6 action, changes that may underlie the procoagulant and proinflammatory milieu of PSVD [26, 27]. Consistent with this, IL‐6–dependent HSC activators such as LIF and CLCF1 were upregulated in parallel with the expanding HSC population and fibrotic burden, supporting IL‐6–mediated HSC activation possibly sustained through a LIF‐driven autocrine loop [28, 29]. This zone‐restricted periportal IL‐6 axis may also explain the characteristic incomplete septal fibrosis of PSVD, in which fibrosis concentrates in zones 1–2 [30, 31]. Although these data are cross‐sectional and stratified by fibrosis stage, precluding conclusions regarding causality or temporal precedence, they consistently implicate IL‐6 signalling in PSVD pathobiology. Given that IL‐6 is already an established therapeutic target in other chronic inflammatory diseases [32], our findings identify IL‐6 trans‐signalling as a rational and actionable target, particularly amenable to repurposing of existing IL‐6 inhibitors for PSVD.

While our study offers valuable insights into PSVD, it has several limitations. First, the retrospective design and relatively small sample size may reduce the statistical power and generalizability of our findings due to potential bias and confounding factors. Specifically, the modest number of liver‐related events precluded comprehensive multivariable adjustment for additional factors (such as comorbidities, concomitant medications, and heterogeneous underlying etiologies), and residual confounding from these variables cannot be excluded. In addition, the transcriptomic subgroup analyses stratified by fibrosis stage and LSVR were based on small numbers of patients per subgroup and are therefore underpowered; the apparent consistency of the signature across subgroups cannot distinguish genuine biological homogeneity from limited statistical power, and these findings should be regarded as exploratory and hypothesis‐generating. However, given the rarity of PSVD, a cohort of 114 patients, including transcriptomic data from 21 individuals, represents a substantial undertaking. Second, as a biomarker, LSVR can be influenced by multiple factors. Spleen volume is not exclusively a reflection of PSVD progression and can be affected by associated conditions, such as myeloproliferative neoplasms. Therefore, the prognostic interpretation of LSVR should be approached with caution in patients with diverse comorbidities, and its validity requires further testing across different clinical settings and subgroups. Third, the transcriptomic subgroup was enriched for patients with more advanced disease, which may skew the results toward more severe cases. Indeed, when we compared the baseline characteristics of the 21 patients selected for RNA sequencing with those of the 93 patients who were not, the sequenced patients had significantly more advanced disease than those not sequenced, including a higher fibrosis stage, lower LSVR, lower platelet count, higher MELD score, and more frequent ascites and portal vein thrombosis. However, this imbalance did not reflect intentional clinical selection but rather the availability of archival FFPE liver tissue of sufficient quantity and quality for RNA sequencing, as patients with advanced disease were more likely to have undergone transplantation, thereby providing adequate tissue for analysis. Given the rarity of PSVD, prospective collection of fresh‐frozen tissue was not feasible, and the FFPE archive was the only realistic source for assembling an 18‐year longitudinal cohort with comprehensive follow‐up. To mitigate these limitations, we applied stringent quality control using a ribo‐depletion protocol optimized for degraded RNA and validated key findings in early‐stage PSVD through immunofluorescence staining of independent human liver tissues and comparisons with previously published datasets. Despite these challenges, the application of advanced analytical methods, including co‐expression network and cell deconvolution analyses, enabled the identification of meaningful prognostic markers and regulatory pathways.

In conclusion, this study identifies LSVR as a valuable prognostic marker for predicting clinical outcomes in PSVD, while also elucidating the regulatory pathways underlying disease progression. By integrating clinical and transcriptomic data, we provide a comprehensive view of the complex interplay between gene expression, cellular composition, and clinical outcomes in PSVD. Notably, IL‐6 trans‐signalling‐induced endotheliopathy in periportal LSECs was associated with proinflammatory and fibrotic changes in PSVD, supporting the possibility that this pathway may represent a potential therapeutic target. Future studies are necessary to validate these findings in larger, prospective cohorts and to explore the therapeutic potential of targeting the identified pathways, particularly those related to the IL‐6 signalling pathway, in order to improve outcomes for patients with PSVD.

## Author Contributions

W.‐M.C. has full access to all the data used in the study and takes responsibility for the integrity of the data and the accuracy of the data analysis. Conception and design: H.‐S.L. and W.‐M.C. Financial support: H.‐S.L. and W.‐M.C. Administrative support: H.‐S.L. and W.‐M.C. Collection and assembly of data: S.H., D.K.Y., Y.Y., I.H.S., and H.J.K. Data analysis and interpretation: All authors Manuscript writing: S.H., D.K.Y., H.‐S.L., and W.‐M.C. Final approval of manuscript: All authors Accountable for all aspects of the work.

## Funding

This work was supported by a grant of the Korea Health Technology R&D Project through the Korea Health Industry Development Institute (KHIDI), funded by the Ministry of Health & Welfare, Republic of Korea (grant number: RS‐2024‐00439637), the Asan Institute for Life Sciences and Corporate Relations of Asan Medical Center (2022IF0017), and the Research Supporting Program of the Korean Association for the Study of the Liver (KASL2021‐03), Republic of Korea to W.‐M.C. Additional support was provided by the National Research Foundation of Korea grant (RS‐2026‐25477096) and an Medical Research Center (MRC) grant (2018R1A5A2020732) to H.‐S.L. funded by the Ministry of Science and ICT, Republic of Korea.

## Disclosure

The funding sources had no role in the design and conduct of the study; collection, management, analysis, and interpretation of data; preparation, review, or approval of the manuscript; and decision to submit the manuscript for publication.

## Conflicts of Interest

The authors declare no conflicts of interest.

## Supporting information


**Figure S1:** Representative CT images of a patient with porto‐sinusoidal vascular disorder (PSVD), overlaid with a red liver mask and green spleen mask generated by the deep learning algorithm. The masks were automatically generated outlining the margins of the liver and spleen, excluding hepatic and splenic vessels as well as any focal hepatic lesions visible on CT images. Liver and spleen volumes were automatically calculated by summing the organ areas on consecutive image slices multiplied by the slice intervals. (A, B) A 57‐year‐old male patient shows a markedly enlarged spleen and para‐oesophageal varices (white arrow), suggestive of portal hypertension. The liver does not show cirrhotic morphology. The deep learning algorithm measured a liver volume of 1055.2 cm^3^ and a spleen volume of 2238.2 cm^3^, resulting in a liver‐to‐spleen volume ratio of 0.47. The patient underwent liver transplantation following the CT scan and was diagnosed with PSVD.
**Figure S2:** PCA and heat map. (A) PCA and heatmap were generated based on 17 421 genes that passed quality control. (B) PCA and the heatmap were created using 1229 DEGs identified between PSVD and HNL samples. DEGs, differentially expressed genes; HNL, histologically normal liver; PCA, principal component analysis; PSVD, porto‐sinusoidal vascular disorder.
**Figure S3:** Comparison of TPM values for marker genes identified by MacParland et al. [12]. (A) The heatmap shows marker gene expression levels (Min–max normalized TPM values) for individual samples, arranged in descending order of LSVR values. An additional clinical parameter (fibrosis stage) is indicated for each sample (Green: periportal LSECs, Blue: Pericentral LSECs, Red: Stellate cells). (B) Boxplots compare TPM values of *FCN2* between HNL and PSVD, as well as across groups based on LSVR and fibrosis stages. The Mann–Whitney *U* test was used for pairwise comparisons, while the Kruskal–Wallis test was applied for three‐group comparisons (*p < 0.05; **p < 0.01; ***p < 0.001). HNL, histologically normal liver; LSECs, liver sinusoidal endothelial cells; LSVR, liver‐to‐spleen volume ratio; PSVD, porto‐sinusoidal vascular disorder; TPM, transcripts per million.
**Figure S4:** Deconvolution analysis of cellular composition. Cell subset proportions in each sample were inferred from single‐cell RNA sequencing data (GSE115469). The analysis performance was assessed using Pearson's correlation (0.81) and the root‐mean‐square error (0.67). Boxplots show the proportion of cell types between HNL and PSVD and across three comparisons: HNL vs. fibrosis stages (F0–2, F3), and HNL vs. LSVR (> 1.33, ≤ 1.33). Mann–Whitney *U* and Kruskal–Wallis tests were used for statistical comparisons. HNL, histologically normal liver; LSVR, liver‐to‐spleen volume ratio; PSVD, porto‐sinusoidal vascular disorder.
**Figure S5:** Gene modules identified through weighted gene co‐expression network analysis (WGCNA). (A) The scale independence value and (B) the mean connectivity are depicted as functions of soft threshold (power). The red line indicates the target range for scale independence from 0.8 to 0.9. (C) Clustering dendrogram of modules with the red line indicating the threshold for merging modules (0.119). (D) Modules are shown before and after merging and clustering. (E) The heatmap displays the correlation coefficients (left box) and corresponding *p* values (right box) between modules (rows) and clinical traits (columns). PSVD is coded as 1 and HNL as 0. Correlation intensity and direction are indicated on the right (red: positive, blue: negative). (F) The bar plot shows the percentage of upregulated genes in PSVD compared to HNL for each module. (G) Boxplots depicts module eigengene values categorized by LSVR (left) and fibrosis stage (right). The Mann–Whitney *U* test was used for two‐group comparisons, and the Kruskal–Wallis test for three‐group comparisons (*p < 0.05; **p < 0.01; ***p < 0.001). HNL, histologically normal liver; LSVR, liver‐to‐spleen volume ratio; PSVD, porto‐sinusoidal vascular disorder.
**Figure S6:** DEGs analysis and GSEA based on fibrosis stage and LSVR in PSVD. (A) The left volcano plot shows seven DEGs between F3 and F0‐2 samples, while the right volcano plot shows 0 DEGs between LSVR ≤ 1.33 and LSVR > 1.33. (B) The left plot shows enrichment results based on fibrosis stage (F3 vs. F0‐2), while the right plot shows enrichment results based on LSVR (≤ 1.33 vs. > 1.33). Significantly enriched Hallmark pathways (FDR < 0.05) are shown in both analyses. (C) Differential gene expression between fibrosis stages. Subgroup analysis revealed seven genes with significant expression differences between F0–2 and F3 stages. DEG, differentially expressed gene; FDR, false discovery rate; GSEA, gene set enrichment analysis; LSVR, liver‐to‐spleen volume ratio; PSVD, porto‐sinusoidal vascular disorder.
**Figure S7:** GSEA based on fibrosis stage and LSVR compared with HNL. (A) Hallmark pathway enrichment in F3 and F0 ~ 2 fibrosis stages compared with HNL. (B) Hallmark pathway enrichment according to LSVR subgroups (≤ 1.33 and > 1.33) compared with HNL. Only significantly enriched pathways (FDR < 0.05) are shown. F, fibrosis stage; FDR, false discovery rate; GSEA, gene set enrichment analysis; HNL, histologically normal liver; LSVR, liver‐to‐spleen volume ratio.
**Figure S8:** ssGSEA of Hallmark pathways identified in PSVD vs. HNL, based on fibrosis stage and LSVR. (A) Heatmap showing ssGSEA scores of selected Hallmark pathways across samples categorized by fibrosis stage and LSVR. (B) Box plots showing ssGSEA scores of representative Hallmark pathways that were significantly different across fibrosis stages based on the Kruskal–Wallis test. (C) Box plots showing ssGSEA scores of representative Hallmark pathways that were significantly different across LSVR subgroups based on Kruskal–Wallis test. (D) GSEA enrichment plots comparing F3 and LSVR ≤ 1.33 relative to HNL. The Mann–Whitney *U* test was used for two‐group comparisons, and the Kruskal–Wallis test for three‐group comparisons (*p < 0.05; **p < 0.01; ***p < 0.001). FDR, false discovery rate; HNL, histologically normal liver; LSVR, liver‐to‐spleen volume ratio; NES, normalized enrichment score; PSVD, porto‐sinusoidal vascular disorder; ssGSEA, single‐sample gene set enrichment analysis.
**Figure S9:** Expression comparison of IL6ST (gp130) dependent cytokines (*IL6*, *LIF*, *OSM*, *IL27*) and IL6 receptors (*IL6R*, *IL6ST*) across three independent datasets. Boxplots show min‐max normalized expression values from our data set (PSVD vs. HNL), GSE77627 (PSVD vs. HNL), and GSE229380 (Selfox vs. Control). HNL, histological normal liver; PSVD, porto‐sinusoidal vascular disorder; Selfox, selenium‐enriched diet plus FOLFOX PSVD rat model.
**Table S1:** Specific and non‐specific histological features of PSVD.
**Table S2:** Univariate analysis of risk factors for liver transplantation.
**Table S3:** Baseline characteristics of participants in the longitudinal cohort.
**Table S4:** Univariate analysis of risk factors for liver‐related death or transplantation.
**Table S5:** Baseline characteristics of patients with versus without RNA sequencing.
**Table S6:** Baseline characteristics of participants in the RNA sequencing study.
**Table S7:** Lists of differentially expressed genes (DEGs).
**Table S8:** Gene Set Enrichment Analysis results for differentially expressed genes.
**Table S9:** List of genes in the darkturquoise module.
**Table S10:** Ingenuity Pathway Analysis (IPA) results for genes in the darkturquoise module. The *p* value was calculated using Fisher's exact test and reflects the probability of an association between the genes in the dataset and the canonical pathways occurring by random chance. The ratio represents the proportion of genes from the darkturquoise module mapped to a given pathway, calculated as the number of module genes in the pathway divided by the total number of genes in the canonical pathway.
**Table S11:** Differentially expressed genes (DEGs) identified in the microarray analysis comparing PSVD samples to HNL samples from the GSE77627 dataset.
**Table S12:** Differentially expressed genes (DEGs) identified in the bulk RNA‐seq analysis comparing Selfox samples to Control samples from the GSE229380 dataset.

## Data Availability

The data that support the findings of this study are available on request from the corresponding author. The data are not publicly available due to privacy or ethical restrictions.
