# Supplementary material for: Identification of Prognostic Factors and Regulatory Pathways in Porto‐Sinusoidal Vascular Disorder
Source: Liver Int. 2026 Sep 30;46(11):e70898. doi: 10.1111/liv.70898 (PMC13625660; doi:10.1111/liv.70898)
Supplement: Supplementary file 1 [file LIV-46-0-s001.zip › liv70898-sup-0001-Supinfo.docx]

**Online Supplementary Materials**

**Identification of prognostic factors and regulatory pathways in porto-sinusoidal vascular disorder**

Subin Heo, Do Kyung Yoon, et al.

**Supplementary Methods**

**Quality Control and Alignment Procedures**

RNA-SeQC (v2.1.2) was used for gene expression quantification.^1^ FastQC (v0.11.9) and MultiQC ensured data quality.^2, 3^ Cutadapt (v3.7) trimmed reads in paired-end mode, filtering adapter sequences and low-quality reads (parameters: '-q 20', '-m 20').^4^ Filtered reads were mapped to the GRCh38 reference genome (GENCODE release 40) using STAR (v2.7.9a) with default settings.^5, 6^ Uniquely mapped reads with mapping quality >255 and fewer than six mismatched bases were selected. RNA-SeQC confirmed unique mapping and rRNA rates, and assessed transcript abundance, expected read counts, and transcripts per million (TPM) for each gene.^1^

**Identification of Differentially Expressed Genes**

Low-expression genes were filtered out by excluding genes with fewer than six reads in more than half of the samples and genes with a normalization value below 0.1 in more than half of the samples. The RNA sequencing data were further refined to include only protein-coding genes. After filtering, gene count and TPM lists were intersected, resulting in the identification of 17,241 genes. Sex- and age-adjusted differential gene expression analysis was performed using the DESeq2 R package.^7^ The cutoff criteria were set at |log_2_ FoldChange (log_2_FC)| >2.0 and a false discovery rate (FDR) < 0.05. Differentially expressed genes (DEGs) were visualized with volcano plots using the EnhancedVolcano (v1.12.0) R package. Additionally, a heatmap was generated using the pheatmap (v1.0.12) R package. Gene set enrichment analysis (GSEA) was conducted using clusterProfiler (v4.2.2) to identify enriched gene sets in PSVD using Hallmark gene sets from the Molecular Signatures Database (MSigDB).^8-10^

**Cell Deconvolution Analysis**

CIBERSORTx was used to estimate cell subset proportions in bulk RNA sequencing data.^11^ The reference matrix for deconvolution was based on single-cell RNA sequencing data from the Gene Expression Omnibus (GSE115469).^12^ Pearson's correlation and root-mean-square error were used to evaluate the accuracy of predicted cell type proportions.

**Weighted Gene Co-Expression Network Analysis**

Co-expression network analysis was performed using WGCNA (v1.72-5).^13^ A scale-free network was constructed with a soft-thresholding power value of 14. Modules were identified using average linkage hierarchical clustering based on the topological overlap matrix. Highly correlated modules were merged using a cutoff value of 0.119. PSVD-related modules were identified through correlation analysis with clinical information (*P* < 0.01). Ingenuity Pathway Analysis (IPA, Qiagen Inc.) was used to identify key canonical pathways in WGCNA gene modules,^14^ with significance determined by Benjamini-Hochberg (BH) corrected overlap p-values.

**Figure S1. Representative CT images of a patient with porto-sinusoidal vascular disorder (PSVD), overlaid with a red liver mask and green spleen mask generated by the deep learning algorithm.** The masks were automatically generated outlining the margins of the liver and spleen, excluding hepatic and splenic vessels as well as any focal hepatic lesions visible on CT images. Liver and spleen volumes were automatically calculated by summing the organ areas on consecutive image slices multiplied by the slice intervals. (A, B) A 57-year-old male patient shows a markedly enlarged spleen and para-esophageal varices (white arrow), suggestive of portal hypertension. The liver does not show cirrhotic morphology. The deep learning algorithm measured a liver volume of 1055.2 cm^3^ and a spleen volume of 2238.2 cm^3^, resulting in a liver-to-spleen volume ratio of 0.47. The patient underwent liver transplantation following the CT scan and was diagnosed with PSVD.

**Figure S2. PCA and heat map.** (A) PCA and heatmap were generated based on 17,421 genes that passed quality control. (B) PCA and the heatmap were created using 1,229 DEGs identified between PSVD and HNL samples. DEGs, differentially expressed genes; HNL, histologically normal liver; PCA, principal component analysis; PSVD, porto-sinusoidal vascular disorder.

**Figure S3. Comparison of TPM values for marker genes identified by MacParland et al.^12^** (A) The heatmap shows marker gene expression levels (Min-max normalized TPM values) for individual samples, arranged in descending order of LSVR values. An additional clinical parameter (fibrosis stage) is indicated for each sample. (Green: periportal LSECs, Blue: Pericentral LSECs, Red: Stellate cells) (B) Boxplots compare TPM values of *FCN2* between HNL and PSVD, as well as across groups based on LSVR and fibrosis stages. The Mann-Whitney U test was used for pairwise comparisons, while the Kruskal-Wallis test was applied for three-group comparisons (**P* < 0.05; ***P* < 0.01; ****P* < 0.001). HNL, histologically normal liver; LSECs, liver sinusoidal endothelial cells; LSVR, liver-to-spleen volume ratio; PSVD, porto-sinusoidal vascular disorder; TPM, transcripts per million.

**Figure S4. Deconvolution analysis of cellular composition.** Cell subset proportions in each sample were inferred from single-cell RNA sequencing data (GSE115469). The analysis performance was assessed using Pearson's correlation (0.81) and the root-mean-square error (0.67). Boxplots show the proportion of cell types between HNL and PSVD and across three comparisons: HNL vs. fibrosis stages (F0–2, F3), and HNL vs. LSVR (>1.33, ≤1.33). Mann-Whitney U and Kruskal-Wallis tests were used for statistical comparisons. HNL, histologically normal liver; LSVR, liver-to-spleen volume ratio; PSVD, porto-sinusoidal vascular disorder.

**Figure S5. Gene modules identified through weighted gene co-expression network analysis (WGCNA).** (A) The scale independence value and (B) the mean connectivity are depicted as functions of soft threshold (power). The red line indicates the target range for scale independence from 0.8 to 0.9. (C) Clustering dendrogram of modules with the red line indicating the threshold for merging modules (0.119). (D) Modules are shown before and after merging and clustering. (E) The heatmap displays the correlation coefficients (left box) and corresponding *P* values (right box) between modules (rows) and clinical traits (columns). PSVD is coded as 1 and HNL as 0. Correlation intensity and direction are indicated on the right (red: positive, blue: negative). (F) The bar plot shows the percentage of upregulated genes in PSVD compared to HNL for each module. (G) Boxplots depicts module eigengene values categorized by LSVR (left) and fibrosis stage (right). The Mann-Whitney U test was used for two-group comparisons, and the Kruskal-Wallis test for three-group comparisons (**P* < 0.05; ***P* < 0.01; ****P* < 0.001). HNL, histologically normal liver; LSVR, liver-to-spleen volume ratio; PSVD, porto-sinusoidal vascular disorder.

**Figure S6. DEGs analysis and GSEA based on fibrosis stage and LSVR in PSVD.** (A) The left volcano plot shows seven DEGs between F3 and F0-2 samples, while the right volcano plot shows 0 DEGs between LSVR ≤1.33 and LSVR >1.33. (B) The left plot shows enrichment results based on fibrosis stage (F3 vs F0-2), while the right plot shows enrichment results based on LSVR (≤1.33 vs >1.33). Significantly enriched Hallmark pathways (FDR < 0.05) are shown in both analyses. (C) Differential gene expression between fibrosis stages. Subgroup analysis revealed seven genes with significant expression differences between F0–2 and F3 stages. DEG, differentially expressed gene; FDR, false discovery rate; GSEA, gene set enrichment analysis; LSVR, liver-to-spleen volume ratio; PSVD, porto-sinusoidal vascular disorder.

**Figure S7. GSEA based on fibrosis stage and LSVR compared with HNL.** (A) Hallmark pathway enrichment in F3 and F0~2 fibrosis stages compared with HNL. (B) Hallmark pathway enrichment according to LSVR subgroups (≤1.33 and >1.33) compared with HNL. Only significantly enriched pathways (FDR < 0.05) are shown. F, fibrosis stage; FDR, false discovery rate; GSEA, gene set enrichment analysis; HNL, histologically normal liver; LSVR, liver-to-spleen volume ratio.

**Figure S8. ssGSEA of Hallmark pathways identified in PSVD vs HNL, based on fibrosis stage and LSVR.** (A) Heatmap showing ssGSEA scores of selected Hallmark pathways across samples categorized by fibrosis stage and LSVR. (B) Box plots showing ssGSEA scores of representative Hallmark pathways that were significantly different across fibrosis stages based on the Kruskal-Wallis test. (C) Box plots showing ssGSEA scores of representative Hallmark pathways that were significantly different across LSVR subgroups based on Kruskal-Wallis test. (D) GSEA enrichment plots comparing F3 and LSVR ≤1.33 relative to HNL. The Mann-Whitney U test was used for two-group comparisons, and the Kruskal-Wallis test for three-group comparisons (**P* < 0.05; ***P* < 0.01; ****P* < 0.001). FDR, false discovery rate; HNL, histologically normal liver; LSVR, liver-to-spleen volume ratio; NES, normalized enrichment score; PSVD, porto-sinusoidal vascular disorder; ssGSEA, single-sample gene set enrichment analysis.

**Figure S9. Expression comparison of IL6ST (gp130) dependent cytokines (*IL6, LIF, OSM, IL27*) and IL6 receptors (*IL6R, IL6ST*) across three independent datasets.** Boxplots show min-max normalized expression values from our data set (PSVD vs HNL), GSE77627 (PSVD vs HNL), and GSE229380 (Selfox vs Control). HNL, histological normal liver; PSVD, porto-sinusoidal vascular disorder; Selfox, selenium-enriched diet plus FOLFOX PSVD rat model.

**Table S1. Specific and non-specific histological features of PSVD**

| **Histologic Features** | **Value (%)** |
| --- | --- |
| **Specific histological signs** |  |
| Obliterative portal venopathy | 58/114 (50.9) |
| Incomplete septal fibrosis | 38/114 (33.3) |
| Nodular regenerative hyperplasia | 21/114 (18.4) |
| **Non-specific histological signs** |  |
| Portal tract vascular abnormalities* | 111/114 (97.4) |
| Architectural disturbance | 61/114 (53.5) |
| Non-zonal sinusoidal dilatation | 50/114 (43.9) |
| Mild perisinusoidal fibrosis | 39/114 (34.2) |

*Including herniated portal vein, hypervascularised portal tracts, and periportal abnormal vessels

**Table S2. Univariate analysis of risk factors for liver transplantation**

| **Variables** | **Univariate analysis** | |
| --- | --- | --- |
|  | **OR (95% CI)** | ***P* value** |
| Varix, *presence* | 3.83 (1.22–11.99) | 0.02 |
| Ascites, *presence* | 6.79 (2.77–16.65) | <0.001 |
| Portal vein thrombosis, *presence* | 6.44 (2.60–16.00) | <0.001 |
| Platelets, *per 10^5^/μL increase* | 0.39 (0.19–0.79) | 0.009 |
| INR, *per 0.01 increase* | 1.06 (1.03–1.08) | <0.001 |
| CTP class  A  B  C | 1 (reference)  6.78 (2.63–17.45)  13.56 (2.16–85.01) | <0.001  0.005 |
| MELD score, *per 1 increase* | 1.26 (1.11–1.43) | <0.001 |
| MELD-Na score, *per 1 increase* | 1.24 (1.11–1.40) | <0.001 |
| Liver-to-spleen volume ratio, *per 1 increase* | 0.28 (0.14–0.54) | <0.001 |
| Fibrosis stage  F0  F1  F2  F3 | 1 (reference)  7.06 (0.81–61.56)  91.43 (10.40–803.96)  360.00 (20.52–6314.85) | 0.08  <0.001  <0.001 |

Abbreviations: CI, confidence interval; CTP, Child-Turcotte-Pugh; INR, international normalized ratio; MELD, model for end-stage liver disease; OR, odds ratio.

**Table S3. Baseline characteristics of participants in the longitudinal cohort.**

| **Characteristics** | **Entire cohort**  **(n = 74)** | **LSVR >1.33**  **(n = 54)** | **LSVR ≤1.33**  **(n = 20)** | ***P* value** |
| --- | --- | --- | --- | --- |
| Age, mean ± SD, y | 45.5 ± 20.8 | 52.0 ± 17.8 | 27.9 ± 20.4 | <0.001 |
| Male sex, n (%) | 44 (56.4) | 28 (51.9) | 15 (75.0) | 0.13 |
| Specific signs of portal hypertension |  |  |  |  |
| Varix, n (%) | 51 (65.4) | 29 (53.7) | 18 (90.0) | 0.009 |
| Variceal bleeding, n (%) | 25 (32.1) | 11 (20.4) | 11 (55.0) | 0.009 |
| Porto-systemic collaterals at imaging | 37 (50.0) | 22 (40.7) | 15 (75.0) | 0.02 |
| Non-specific signs of portal hypertension |  |  |  |  |
| Platelets < 150 x 10^3^/μL | 49 (66.2) | 30 (55.6) | 19 (95.0) | 0.004 |
| Ascites, n (%) | 16 (20.5) | 12 (22.2) | 3 (15.0) | 0.72 |
| Spleen size ≥ 13 cm in largest axis | 37 (50.0) | 20 (37.0) | 17 (85.0) | 0.001 |
| Encephalopathy, n (%) | 3 (3.8) | 2 (3.7) | 0 (0.0) | 0.95 |
| Portal vein thrombosis, n (%) | 22 (28.6) | 9 (17.0) | 11 (55.0) | 0.003 |
| Chronic HBV infection, n (%) | 1 (1.3) | 1 (1.9) | 0 (0.0) | >0.99 |
| Associated conditions / Drug |  |  |  |  |
| Immunological disorder, n (%) | 4 (5.1) | 4 (7.4) | 0 (0.0) | 0.50 |
| Hematological disorder, n (%) | 4 (5.1) | 3 (5.6) | 1 (5.0) | >0.99 |
| Prothrombotic disorder, n (%) | 18 (23.1) | 15 (27.8) | 3 (15.0) | 0.41 |
| Drugs, n (%) | 4 (5.1) | 3 (5.6) | 1 (5.0) | >0.99 |
| Genetic disorder, n (%) | 1 (1.3) | 0 (0.0) | 1 (5.0) | 0.60 |
| HIV infection, n (%) | 1 (1.3) | 1 (1.9) | 0 (0.0) | >0.99 |
| Laboratory values |  |  |  |  |
| AST, median (IQR), IU/L | 26 (21, 36) | 26 (20, 36) | 27 (24, 34) | 0.30 |
| ALT, median (IQR), IU/L | 21 (14, 34) | 21 (13, 36) | 21 (16, 33) | 0.90 |
| Albumin, median (IQR), g/dL | 3.6 (3.1, 3.9) | 3.5 (3.1, 3.9) | 3.8 (3.4, 4.0) | 0.18 |
| Total bilirubin, median (IQR), mg/dL | 0.8 (0.5, 1.1) | 0.7 (0.5, 0.9) | 1.1 (0.8, 2.1) | <0.001 |
| Platelets, median (IQR), x1000/μL | 107 (67, 196) | 141 (100, 203) | 54 (41, 74) | <0.001 |
| INR, median (IQR) | 1.11 (1.03, 1.19) | 1.06 (1.01, 1.15) | 1.22 (1.18, 1.37) | <0.001 |
| Creatinine, median (IQR), mg/dL | 0.7 (0.6, 0.9) | 0.7 (0.6, 0.9) | 0.8 (0.5, 0.9) | 0.30 |
| White blood cells, median (IQR), x10^3^/μL | 4.2 (3.2, 6.0) | 4.4 (3.8, 6.6) | 3.4 (2.5, 4.1) | 0.001 |
| Hemoglobin, median (IQR), g/dL | 12.1 (9.7, 13.2) | 12.1 (9.7, 13.5) | 12.2 (9.5, 13.1) | 0.66 |
| CTP class, n (%)  A  B  C | 60 (76.9)  17 (21.8)  1 (1.3) | 42 (77.8)  11 (20.3)  1 (1.9) | 16 (80.0)  4 (20.0)  0 (0.0) | 0.83 |
| MELD score, mean ± SD | 8 (7, 9) | 8 (7, 8) | 10 (9, 12) | <0.001 |
| MELD-Na score, mean ± SD | 9 (8, 11) | 9 (8, 10) | 12 (10, 12) | <0.001 |
| Liver stiffness, median (IQR), kPa* | 7.7 (5.0, 11.6) | 6.9 (4.7, 11.5) | 9.2 (6.6, 13.4) | 0.26 |
| Liver volume, median (IQR), cm^3^ | 1,086 (880, 1,284) | 1,116 (880, 1,300) | 1,018 (869, 1,260) | 0.30 |
| Spleen volume, median (IQR), cm^3^ | 485 (286, 759) | 390 (262, 595) | 1,110 (832, 1,568) | <0.001 |
| LSVR, median (IQR) | 2.01 (1.28, 3.80) | 2.96 (1.95, 4.62) | 0.88 (0.71, 1.16) | <0.001 |
| METAVIR fibrosis score, n (%)  F0  F1  F2 | 39 (50.0)  32 (41.0)  7 (9.0) | 24 (44.4)  26 (48.2)  4 (7.4) | 13 (65.0)  4 (20.0)  3 (15.0) | 0.08 |

Abbreviations: ALT, alanine aminotransferase; AST, aspartate aminotransferase; CTP, Child-Turcotte-Pugh; HBV, hepatitis B virus; INR, international normalized ratio; IQR, interquartile range; LSVR, liver-to-spleen volume ratio; MELD, model for end-stage liver disease; SD, standard deviation.

^†^Including herniated portal vein, hypervascularised portal tracts, and periportal abnormal vessels.

^*^Missing data on liver stiffness for 28 patients.

**Table S4. Univariate analysis of risk factors for liver-related death or transplantation.**

| **Variables** | **Univariate analysis** | |
| --- | --- | --- |
|  | **HR (95% CI)** | ***P* value** |
| Age, *per 1-year increase* | 1.01 (0.98–1.04) | 0.50 |
| Ascites, *presence* | 1.51 (0.40–5.65) | 0.54 |
| Portal vein thrombosis, *presence* | 0.94 (0.28–3.15) | 0.93 |
| Platelets, *per 10^5^/μL increase* | 0.36 (0.11–1.15) | 0.09 |
| INR, *per 0.1 increase* | 1.35 (0.95–1.92) | 0.09 |
| CTP class B or C | 2.08 (0.61–7.03) | 0.24 |
| MELD score, *per 1 increase* | 1.14 (0.98–1.33) | 0.08 |
| MELD-Na score, *per 1 increase* | 1.14 (0.99–1.32) | 0.08 |
| **Liver-to-spleen volume ratio, *per 1 increase*** | **0.37 (0.15–0.90)** | **0.03** |
| Fibrosis stage  F0  F1  F2 | 1 (reference)  1.84 (0.53–6.42)  1.95 (0.22–16.93) | 0.34  0.55 |

Abbreviations: CI, confidence interval; CTP, Child-Turcotte-Pugh; HR, hazard ratio; INR, international normalized ratio; MELD, model for end-stage liver disease.

**Table S5. Baseline characteristics of patients with versus without RNA sequencing**

| **Characteristics** | **No RNA-seq**  **(n = 93)** | **RNA-seq**  **(n = 21)** | ***P* value** |
| --- | --- | --- | --- |
| Age, mean ± SD, y | 46.4 ± 20.0 | 48.1 ± 15.3 | 0.71 |
| Male sex, n (%) | 52 (55.9) | 11 (52.4) | 0.96 |
| Varix, n (%) | 62 (66.7) | 19 (90.5) | 0.06 |
| Variceal bleeding, n (%) | 27 (29.0) | 9 (42.9) | 0.33 |
| Ascites, n (%) | 26 (28.0) | 13 (61.9) | 0.007 |
| Encephalopathy, n (%) | 5 (5.4) | 2 (9.5) | 0.83 |
| Portal vein thrombosis, n (%) | 30 (32.6) | 16 (76.2) | 0.001 |
| Chronic HBV infection, n (%) | 3 (3.2) | 0 (0.0) | 0.94 |
| Associated conditions / Drugs |  |  |  |
| Immunological disorder, n (%) | 4 (4.3) | 0 (0.0) | 0.76 |
| Hematological disorder, n (%) | 5 (5.4) | 1 (4.8) | >0.99 |
| Prothrombotic disorder, n (%) | 21 (22.6) | 4 (19.0) | 0.95 |
| Drugs, n (%) | 4 (4.9) | 0 (0.0) | 0.76 |
| Genetic disorder, n (%) | 1 (1.2) | 0 (0.0) | >0.99 |
| HIV infection, n (%) | 1 (1.2) | 0 (0.0) | >0.99 |
| Laboratory values |  |  |  |
| AST, median (IQR), IU/L | 26 (21, 36) | 24 (19, 27) | 0.08 |
| ALT, median (IQR), IU/L | 21 (13, 28) | 16 (13, 18) | 0.03 |
| Albumin, median (IQR), g/dL | 3.5 (3.1, 3.9) | 3.1 (2.8, 3.5) | 0.004 |
| Total bilirubin, median (IQR), mg/dL | 0.8 (0.6, 1.2) | 1.5 (0.7, 2.4) | 0.008 |
| Platelets, median (IQR), x10^3^/μL | 103 (64, 190) | 48 (41, 101) | 0.001 |
| INR, median (IQR) | 1.13 (1.04, 1.24) | 1.32 (1.19, 1.48) | <0.001 |
| Creatinine, median (IQR), mg/dL | 0.7 (0.6, 0.9) | 0.7 (0.5, 0.8) | 0.53 |
| White blood cells, median (IQR), x10^3^/μL | 4.1 (3.1, 5.7) | 3.4 (2.7, 4.1) | 0.03 |
| Hemoglobin, median (IQR), g/dL | 11.1 (9.5, 13.1) | 9.3 (8.2, 11.0) | 0.002 |
| CTP class, n (%)  A  B  C | 64 (68.8)  25 (26.9)  4 (4.3) | 6 (28.6)  13 (61.9)  2 (9.5) | 0.003 |
| MELD score, mean ± SD | 8 (7, 11) | 13 (9, 14) | <0.001 |
| MELD-Na score, mean ± SD | 10 (8, 12) | 15 (10, 16) | <0.001 |
| Liver stiffness, median (IQR), kPa* | 7.5 (4.9, 11.6) | 9.9 (5.7, 10.8) | 0.73 |
| Liver volume, median (IQR), cm^3^* | 1,065 (810, 1,270) | 1,011 (580, 1,348) | 0.45 |
| Spleen volume, median (IQR), cm^3^* | 545 (295, 856) | 845 (631, 1,585) | 0.003 |
| Liver-to-spleen volume ratio, median (IQR)* | 1.94 (1.13, 3.44) | 0.88 (0.63, 1.28) | <0.001 |
| METAVIR fibrosis score, n (%)  F0  F1  F2  F3 | 39 (41.9)  38 (40.9)  11 (11.8)  5 (5.4) | 2 (9.5)  2 (9.5)  12 (57.1)  5 (23.8) | <0.001 |

Abbreviations: ALT, alanine aminotransferase; AST, aspartate aminotransferase; CTP, Child-Turcotte-Pugh; HBV, hepatitis B virus; INR, international normalized ratio; IQR, interquartile range; MELD, model for end-stage liver disease; SD, standard deviation.

^*^Missing data on liver stiffness for 61 patients, and on liver, spleen volume, and liver-to-spleen volume ratio for 4 patients.

**Table S6. Baseline characteristics of participants in the RNA sequencing study.**

| **Characteristics** | **HNL group**  **(n = 6)** | **PSVD**  **(n = 21)** | ***P* value** |
| --- | --- | --- | --- |
| Age, mean ± SD, y | 58.5 ± 5.7 | 44.5 ± 17.8 | 0.07 |
| Male sex, n (%) | 3 (50.0) | 10 (47.6) | 0.60 |
| AST, median (IQR), IU/L | 19 (16, 25) | 24 (19, 28) | 0.41 |
| ALT, median (IQR), IU/L | 16 (12, 20) | 15 (12, 22) | 0.88 |
| Albumin, median (IQR), g/dL | 3.7 (3.2, 4.0) | 3.1 (2.8, 3.6) | 0.37 |
| Total bilirubin, median (IQR), mg/dL | 0.5 (0.3, 0.8) | 1.3 (0.9, 2.1) | 0.01 |
| Platelets, median (IQR), x1000/μL | 259 (198, 273) | 48 (41, 93) | <0.001 |
| INR, median (IQR) | 0.96 (0.94, 1.00) | 1.32 (1.18, 1.42) | <0.001 |
| Creatinine, median (IQR), mg/dL | 0.7 (0.6, 0.8) | 0.7 (0.5, 0.8) | 0.95 |
| White blood cells, median (IQR), x10^3^/μL | 5.6 (4.4, 6.0) | 3.3 (2.4, 3.8) | 0.03 |
| Hemoglobin, median (IQR), g/dL | 12.7 (10.6, 13.1) | 8.9 (8.2, 11.0) | 0.03 |
| MELD score, mean ± SD | 6 (6, 8) | 12 (9, 14) | 0.001 |
| MELD-Na score, mean ± SD | 7 (6, 8) | 13 (10, 15) | 0.001 |
| Liver stiffness, median (IQR), kPa* | – | 9.9 (5.7, 10.8) | – |
| Liver volume, median (IQR), cm^3^ | 1,365 (918, 1,448) | 1,017 (581, 1,348) | 0.18 |
| Spleen volume, median (IQR), cm^3^ | 164 (114, 186) | 959 (692, 1,585) | <0.001 |
| Liver-to-spleen volume ratio, median (IQR) | 7.48 (7.23, 12.16) | 0.84 (0.51, 1.28) | <0.001 |
| METAVIR fibrosis score, n (%)  F0  F1  F2  F3 | 6 (100.0)  0 (0.0)  0 (0.0)  0 (0.0) | 1 (4.8)  3 (14.3)  10 (47.6)  7 (33.3) | 0.001 |

Abbreviations: ALT, alanine aminotransferase; AST, aspartate aminotransferase; HBV, hepatitis B virus; INR, international normalized ratio; IQR, interquartile range; MELD, model for end-stage liver disease; SD, standard deviation.

**Table S7. Lists of differentially expressed genes (DEGs).**

*Data provided in a separate file*

**Table S8. Gene Set Enrichment Analysis results for differentially expressed genes.**

*Data provided in a separate file*

**Table S9. List of genes in the darkturquoise module.**

*Data provided in a separate file*

| **Ingenuity Canonical Pathways** | **FDR** | **Ratio** | **Overlaps with dataset** | **Molecules** |
| --- | --- | --- | --- | --- |
| Smooth Muscle Contraction | 0.03715 | 0.093 | 4/43 (9%) | *ACTG2, ANXA1, LMOD1, TPM2* |
| Cardiac Hypertrophy Signaling (Enhanced) | 0.03715 | 0.0208 | 11/529 (2%) | *ADRA2C, CACNA2D1, FGF2, FGF7, IL6, IL6R, LIF, PDE10A, PDE1A, PLCE1, PRKG1* |
| Interleukin-6 family signaling | 0.03715 | 0.125 | 3/24 (13%) | *IL6, IL6R, LIF* |
| Interleukin-4 and Interleukin-13 signaling | 0.03715 | 0.0455 | 5/110 (5%) | *ANXA1, FGF2, IL6, IL6R, LIF* |
| Wound Healing Signaling Pathway | 0.03715 | 0.0294 | 7/238 (3%) | *COL15A1, COL21A1, FGF2, FGF7, IL6, LAMA3, LIF* |
| Airway Pathology in Chronic Obstructive Pulmonary Disease | 0.03715 | 0.0442 | 5/113 (4%) | *APOD, FGF2, FGF7, IL6, LIF* |

**Table S10. Ingenuity Pathway Analysis (IPA) results for genes in the darkturquoise module**. The *P* value was calculated using Fisher's exact test and reflects the probability of an association between the genes in the dataset and the canonical pathways occurring by random chance. The ratio represents the proportion of genes from the darkturquoise module mapped to a given pathway, calculated as the number of module genes in the pathway divided by the total number of genes in the canonical pathway.

Abbreviations: FDR, false discovery rate.

**Table S11. Differentially expressed genes (DEGs) identified in the microarray analysis comparing PSVD samples to HNL samples from the GSE77627 dataset.**

*Data provided in a separate file*

**Table S12. Differentially expressed genes (DEGs) identified in the bulk RNA-seq analysis comparing Selfox samples to Control samples from the GSE229380 dataset.**

*Data provided in a separate file*

**REFERENCES**

1.	DeLuca DS, Levin JZ, Sivachenko A, et al. RNA-SeQC: RNA-seq metrics for quality control and process optimization. Bioinformatics. 2012;28(11):1530-2. Epub 20120425.

2.	Andrews S. FastQC: A Quality Control Tool for High Throughput Sequence Data. 2010 [cited 2023 February 12]; Available from: <https://www.bioinformatics.babraham.ac.uk/projects/fastqc/>.

3.	Ewels P, Magnusson M, Lundin S, et al. MultiQC: summarize analysis results for multiple tools and samples in a single report. Bioinformatics. 2016;32(19):3047-8. Epub 20160616.

4.	Martin M. Cutadapt removes adapter sequences from high-throughput sequencing reads. EMBnetjournal. 2011;17(1):3. Epub 2011-08-02.

5.	Dobin A, Davis CA, Schlesinger F, et al. STAR: ultrafast universal RNA-seq aligner. Bioinformatics. 2013;29(1):15-21. Epub 20121025.

6.	Frankish A, Carbonell-Sala S, Diekhans M, et al. GENCODE: reference annotation for the human and mouse genomes in 2023. Nucleic Acids Res. 2023;51(D1):D942-D9.

7.	Love MI, Huber W, Anders S. Moderated estimation of fold change and dispersion for RNA-seq data with DESeq2. Genome Biol. 2014;15(12):550.

8.	Yu G, Wang LG, Han Y, et al. clusterProfiler: an R package for comparing biological themes among gene clusters. OMICS. 2012;16(5):284-7. Epub 20120328.

9.	Liberzon A, Birger C, Thorvaldsdottir H, et al. The Molecular Signatures Database (MSigDB) hallmark gene set collection. Cell Syst. 2015;1(6):417-25.

10.	Subramanian A, Tamayo P, Mootha VK, et al. Gene set enrichment analysis: a knowledge-based approach for interpreting genome-wide expression profiles. Proc Natl Acad Sci U S A. 2005;102(43):15545-50. Epub 20050930.

11.	Steen CB, Liu CL, Alizadeh AA, et al. Profiling Cell Type Abundance and Expression in Bulk Tissues with CIBERSORTx. Methods Mol Biol. 2020;2117:135-57.

12.	MacParland SA, Liu JC, Ma XZ, et al. Single cell RNA sequencing of human liver reveals distinct intrahepatic macrophage populations. Nat Commun. 2018;9(1):4383. Epub 2018/10/24.

13.	Langfelder P, Horvath S. WGCNA: an R package for weighted correlation network analysis. BMC Bioinformatics. 2008;9:559. Epub 20081229.

14.	Kramer A, Green J, Pollard J, Jr., et al. Causal analysis approaches in Ingenuity Pathway Analysis. Bioinformatics. 2014;30(4):523-30. Epub 20131213.
